# Exploring changes in family routines and family quality of life among Israeli families during the COVID-19 pandemic

**DOI:** 10.7717/peerj.19587

**Published:** 2025-07-03

**Authors:** Liat Hen-Herbst, Yael Fogel

**Affiliations:** Department of Occupational Therapy, Ariel University, Ariel, Israel

**Keywords:** Family well-being, Coronavirus pandemic, Routines, Family interactions

## Abstract

**Background:**

The COVID-19 pandemic has significantly disrupted family life globally, altering daily routines and impacting family quality of life (FQOL). This study explores the changes in family routines during the pandemic lockdowns and their correlation with FQOL.

**Methods:**

Participants included 253 families with children aged 3 to 18, recruited through an online survey. Families completed the Family Routines Inventory and the FQOL Scale, reporting on both pre-pandemic and during-pandemic conditions. Data analysis included principal component analysis and regression analysis to identify changes in routine frequency and importance and their predictive relationship with FQOL.

**Results:**

The principal component analysis resulted in three distinct factors of family routines, highlighting significant shifts in the frequency and importance of these routines from before and during COVID-19. Notably, routines involving within-family interactions increased, whereas those involving external interactions decreased. Regression analysis showed that the frequency of within-family interactions (Factor 1) and the importance of general morning and evening routines (Factor 3) significantly predicted FQOL, accounting for 24% of the variance in FQOL scores during the pandemic.

**Conclusion:**

The COVID-19 pandemic has underscored the critical role of family routines in maintaining FQOL in times of crisis. Interventions aimed at supporting families should consider enhancing the adaptability and resilience of family routines to foster well-being during unpredictable stressors. Future research should continue to explore the dynamics of family routines and their impact on FQOL across different types of crises.

## Introduction

In 2020, the COVID-19 pandemic spread rapidly, profoundly affecting individuals and families worldwide and resulting in unprecedented changes. Families with children faced numerous challenges as they adjusted to the disruptions caused by school closures, home-based learning, reduced outdoor activities, stay-at-home orders, quarantines, and parents’ changing work schedules (Chen, Byrne & Vélez, 2022; Coyle et al., 2023). The lack of regular routines and the social isolation imposed during lockdowns exerted notable effects on the physical and psychological well-being of children (Singh et al., 2020; Wang et al., 2021) and adults (Girma et al., 2024; Jurblum, Ng & Castle, 2020).

Research investigating the consequences of COVID-19 pandemic lockdowns has indicated a rise in children’s behavioral issues (Liu et al., 2021), sleep disturbances (Jiao et al., 2020), irritability, inattention, and clingy behavior in all age groups (Viner et al., 2020). Although the pandemic originated outside the family system, many parents and children likely perceive it as a family stressor, given the novelty and uncertainty concerning this disease. Families worldwide are collectively experiencing a new range of stressors that threaten their health, safety, and well-being (Brown et al., 2020). Studies showed that, compared to adults without children, parents experienced higher stress during COVID-19 (*e.g*., Adams et al., 2021). This heightened stress is attributed to the additional difficulties, such as supervising their children’s remote schooling, disruptions in extracurricular activities, and the complexities of helping children cope with feelings of uncertainty and adjustment (American Psychological Association, 2020).

Whereas many unpredictable changes in families are not easily modifiable (*e.g*., losing a job), family routines can be considered a fundamental aspect of the family environment amenable to adjustment (Glynn et al., 2021). Routines are structured sequences of occupations or activities that organize daily life and can serve as either protective or risk factors for health and well-being (Fiese et al., 2002; Koome, Hocking & Sutton, 2012). Embedded in cultural and ecological contexts (Koome, Hocking & Sutton, 2012), routines can be considered fixed sequences of typical daily events; they provide predictability in the environment and may help establish appropriate behavior (Milan et al., 1981; Sytsma, Kelley & Wymer, 2001). Despite requiring momentary time commitment, they add to long-term emotional investment (Koome, Hocking & Sutton, 2012). Family routines involve at least two family members, typically a parent and a child, and are carried out consistently and predictably, such as family mealtimes and bedtime rituals (Fiese et al., 2002).

Research has suggested that children are less physically active when not in school, have poorer sleep hygiene, and spend more time in front of screens (Brazendale et al., 2017). With the newly changing structures of daily life activities come increased demands on parents to develop new routines flexibly (Prime, Wade & Browne, 2020). Past research investigating family resilience (*i.e*., successful coping in adversity enabling the family to flourish) identified regular family routines as a protective coping resource in potentially stressful situations (Black & Lobo, 2008) and as embedded within the cultural and ecological contexts of families’ lives (Spagnola & Fiese, 2007). Different routines are distinguished by their concrete goals, such as preparing and eating a meal, readying a child for bed, or having “family time” together each week (Howe, 2002). Thus, regular family routines are associated with parental competence, improved child health, parent–child harmony, children’s academic achievements, emotional climates supporting child development (Spagnola & Fiese, 2007), and positive family well-being (McCubbin & McCubbin, 1988). Routines can improve family stability (De Goede & Greeff, 2016).

In families’ daily lives, activities at mealtimes, mornings, and evenings can be settings for routines that provide family members with structure and feelings of security (Crespo et al., 2013; Rodger & Umaibalan, 2011). According to Kliewer & Kung (1998), family cohesion and routines may also be a protective buffer against environmental stressors. Maintaining family routines can protect the children’s development and well-being, even in situations marked by increased risk (Fiese et al., 2002; Kiser et al., 2005). Further, when the whole family participates in specific routines, each member is more likely to engage in health-promoting behaviors (Crespo et al., 2013; Sussman et al., 2016).

Matias, Dominski & Marks (2020) explored human needs during the COVID-19 isolation and documented that unmet self-protection needs become “normal” during lockdowns. Individuals experience systematic frustration in a deep-seated need to protect themselves and their families. This high frustration creates fear, anxiety, and distress because individuals feel incompetent to guarantee the safety of their loved ones, family, and self.

Although environmental stressors might amplify the likelihood of children experiencing mental health challenges, a diverse range of protective elements can help them uphold or enhance their well-being in uncertain and adverse conditions (Sheen et al., 2021). Building on the original quality of life literature, researchers conceptualized family quality of life (FQOL) as similarly multidimensional (Poston et al., 2003; Samuel, Tarraf & Marsack, 2018). This is “a dynamic sense of well-being of the family, collectively and subjectively defined and informed by its members, in which individual- and family-level needs interact” (Zuna, Turnbull & Summers, 2009, p. 262). Closely related to the concept of family well-being, FQOL has been predominantly investigated within the context of families of individuals with disabilities (Brown & Schippers, 2016). It encompasses several key domains, including emotional well-being, family and interpersonal relationships, material well-being, personal development, physical well-being, disability-related support, social inclusion, and rights (Geprägs et al., 2024). Its central focus comes in light of high-quality evidence that children’s adjustment largely depends on the general climate and relationships within a family (Brown, 2017). Additionally, interventions to support child well-being are more effective when they include family components (Haine-Schlagel & Walsh, 2015).

The COVID-19 pandemic significantly affected the home environment and family functioning. This period provided a unique opportunity for families to embrace routines. Suddenly eliminating other external factors (*e.g*., school, work, and extracurricular activities) placed the family environment at the core of daily life (Bates et al., 2021). Staying at home may have also offered parents unique opportunities to establish more flexible family routines in ways that were previously inaccessible (Cito et al., 2020). Nevertheless, the increased demands in parenting responsibilities (*e.g*., homeschooling) during the COVID-19 pandemic may have impeded parents’ ability to engage with consistent parenting strategies (*e.g*., planning, monitoring, and limit-setting) and adversely affected the family’s daily function. Engaging in family routines during the COVID-19 pandemic seemed to buffer the impact of stress on family resilience (Bates et al., 2021). Furthermore, parental inflexibility was linked to reduced family cohesion and constructive parenting strategies and increased family discord (Daks, Peltz & Rogge, 2020).

The family, an enduring social institution, is the essential building block of human societies. The family setting holds substantial potential as a protective shield for children’s behaviors and mental well-being (Robinson & Neece, 2015). Within humanitarian crisis scenarios, parents assume a vital responsibility for safeguarding their children’s mental health (Panter-Brick et al., 2011; Power et al., 2016), and adept parenting can shield children during periods of risk, disruption, and unpredictability (Bell, Flay & Paikoff, 2002). Specifically, researchers of the COVID-19 pandemic indicated that the impacts of lockdown and the factors that protected well-being during lockdown should be considered beyond the individual level—they should, at least, include the family’s role as a protective factor in managing the lockdown effects (Sheen et al., 2021).

Israel represents a unique cultural context characterized by strong family-oriented values, collectivist cultural traits, and community resilience mechanisms shaped significantly by ongoing geopolitical challenges and security threats. These characteristics position Israeli families distinctively when navigating crises compared to other cultural contexts. Previous research underscored the resilience embedded within Israeli families, which demonstrated a robust capacity for coping with stress and uncertainty through communal and familial support networks (Shamai & Kimhi, 2007; Slone & Mann, 2016). Consequently, examining how Israeli families adjust their routines and preserve their FQOL during the unprecedented global crisis of COVID-19 provides critical insights into adaptive coping mechanisms and resilience processes relevant beyond the immediate pandemic context.

Moreover, Israel’s governmental and societal response to COVID-19, including strict lockdowns, rapid vaccination campaigns, and mandatory quarantine policies, uniquely influenced family routines and daily life compared to other nations. Such distinctive measures and their societal compliance or resistance provide fertile ground for exploring how enforced isolation, disruption of established routines, and the ensuing adaptive strategies shape family dynamics and quality of life within this cultural milieu (Kimhi et al., 2020; Bodas & Peleg, 2020). Hence, investigating family adjustments, specifically among Israeli families, contributes to understanding cultural variability in coping with crises and aids in developing culturally sensitive policy recommendations and interventions to enhance family resilience and well-being during similar future challenges.

The disruptions to families’ daily lives brought about by the COVID-19 pandemic offer a chance to explore the potential advantages of family routines in a period characterized by stress and uncertainty. Although previous literature examined the importance of family routines generally, a notable gap exists regarding how such routines change and impact FQOL during global crises like COVID-19. This study uniquely addresses this gap by examining routine adjustments explicitly linked to the pandemic. Accordingly, the primary research question guiding this study is: “What specific changes in the frequency and perceived importance of family routines during the first COVID-19 lockdown are associated with variations in family quality of life (FQOL)”. Specifically, this study’s questions are: (1) How did the frequency and importance of specific family routines change from before to during the first COVID-19 lockdown? (2) What are the specific relationships between these family routine changes (frequency and importance) and FQOL during the first COVID-19 lockdown? (3) To what extent can family routine frequency and importance during lockdown predict the overall FQOL families experienced during the first COVID-19 lockdown?

This study investigates how COVID-19 affected family routines and how these changes relate to family quality of life. Although previous studies have examined pandemic stress broadly, there is a lack of research on the specific role and impact of changes in family routines. This research fills that gap by focusing on these routine changes among Israeli families during the first COVID-19 lockdown.

## Materials and Methods

### Study design

A cross-sectional quantitative research design was employed to investigate changes in family routines and their associations with family quality of life (FQOL) during the initial COVID-19 lockdown.

### Participants

We recruited participants through the MIDGAM survey company (http://www.midgampanel.com/research/en/index.asp), which emailed invitations to potential candidates. Parents who indicated a willingness to participate in exchange for monetary reimbursement accessed the online questionnaire through a Qualtrics link. The inclusion criterion was families with children between 3 and 18 years old. We defined *family* based on the Beach Center on Disability’s (2015) definition—individuals closely involved in the household’s day-to-day affairs and who support each other regularly, regardless of whether related by marriage, blood, or a close personal relationship.

To detect medium effects, we used G*Power to determine the sample size with a statistical power of 95% and alpha of 0.05, accounting for up to 15 predictors. This calculation indicated a minimum sample size of 115 participants. Ultimately, the sample consisted of 253 families with children 18 years and younger. Each family had one parent (58.5% mothers) who signed online consent forms. We surveyed between April 30 and May 24, 2020. This period coincided with an Israeli government stay-at-home order (March 14, 2020–May 27, 2020) restricting travel and work to essential needs like obtaining food or medicine (Last, 2020). The mean age of the 253 parents who completed self-report questionnaires was 37.49 years (*SD* = 6.88). Of the respondents, 41.5% were fathers, and 72.3% had an academic education of a bachelor’s degree or higher. Additionally, 63.2% of the families reported an income above the average monthly salary of 12,622 NIS (Israel Central Bureau of Statistics, 2023), and most (73.1%) resided in urban areas. The mean number of children per family was 2.90 (*SD* = 1.12). (See Fogel, Sela & Hen-Herbst (2022) for additional sample characteristic details.)

### Measures

#### Demographic questionnaire

The online demographic questionnaire developed for this study included parents’ ages, roles, and education (high school, vocational, or academic), family income level, number of children in the family, and place of residence.

#### Family routines inventory

The Family Routines Inventory (FRI; Jensen et al., 1983) lists 28 routines in the daily lives of families with at least one child. Participants indicated on a four-point Likert scale from 1 (*rarely*) to 4 (*always/every day*) the frequency of each routine in their family. Then, participants ranked the routine’s importance to keeping the family firm as a unit on a three-point Likert scale from 1 (*not important*) to 3 (*very important*). We have permission to use this instrument from the copyright holders (Elsevier). Cronbach’s alphas were 0.83 and 0.81 for the FRI routine frequency and importance before and during COVID-19, respectively.

#### FQOL scale

The FQOL Scale (Hoffman et al., 2006) assesses parental perception of FQOL. It contains 25 items in four subscales—family interaction (*e.g*., *M. family enjoys spending time together*), parenting (*e.g*., *M. family members help the children with schoolwork and activities*), emotional well-being (*e.g*., *M. family members have friends or others who provide support*), and physical and material well-being (*e.g*., *M. family members have transportation to get to the places they need to be*). It assesses functioning and cohesion in families with and without disabled family members (Summers et al., 2007; Zuna, Turnbull & Summers, 2009). For this study, we removed four questions regarding disability-related support because they would not necessarily apply to all participants. Studies of families without disabilities have confirmed the modified FQOL Scale’s validity and reliability (*e.g*., Zuna, Turnbull & Summers, 2009). Participants indicated their satisfaction with each item on a five-point Likert scale from 1 (*very unsatisfied*) to 5 (*very satisfied*). Higher scores indicated greater satisfaction and a better perception of FQOL overall. We have permission to use this instrument from the copyright holders (Beach Center on Disability). The Cronbach’s alphas in the current study for the 21 items before and during COVID-19 were 0.94 and 0.95, respectively.

### Procedure

The Ethics Committee of Ariel University approved the study (No. AU-HEAY20200405). We followed ethical standards throughout the study. Participants signed an online informed consent form describing the research aims and its voluntary frame. Only the recruiting company knew the participants’ identities; hence, all the participants were anonymous to the researchers, ensuring privacy. Participants received an agreed payment from the recruiting company upon completion. One parent of each family received a link to the FRI, FQOL Scale, and demographic questionnaire. Participants coded the FRI and FQOL Scale twice—once for describing their family before the pandemic (pre-COVID-19) and again regarding their family during the crisis’s first lockdown (during-COVID-19).

### Data analysis

We used IBM SPSS (Version 25.0) to analyze the data. The descriptive statistics for demographic characteristics used means, standard deviations, and ranges for continuous variables and frequencies for discrete variables. Before extracting factors, we used a Kaiser-Meyer-Olkin (KMO) measure of sampling adequacy to assess the respondent data’s suitability for factor analysis. The KMO index ranges from 0 to 1, with 0.60 considered suitable (Kaiser & Rice, 1974). In this study, the KMO index was 0.87, and the Bartlett test of sphericity yielded *p* < 0.001, indicating suitability for factor analysis.

We performed a principal component analysis (PCA) with principal components extraction and varimax rotation to examine the FRI (Altman, 1991). We eliminated items that yielded a factor-loading value of less than 0.3 from the pool. The factorial structure and final item set in each factor were subjected to a revised PCA without the failed items. We assessed differences in dependent-variable levels before and during COVID-19 using paired *t*-tests. We used Pearson correlations to determine relationships among dependent variables and stepwise regression for significant predictors of total well-being for the first COVID-19 lockdown variable. Testing whether the data met the collinearity assumption indicated that multicollinearity was not a concern (FRI Factor 1, tolerance = 0.77, Variance Inflation Factor = 1.29; FRI Factor 3, tolerance = 0.77, Variance Inflation Factor = 1.29).

## Results

### Family routine frequency and importance changes before and during COVID-19

In the initial stage, we conducted a PCA on pre-COVID-19 data, which led to removing four items from the FRI due to low factor loadings. This resulted in a refined scale with 24 items organized into four distinct factors. Subsequently, we conducted a revised PCA on the updated scale. This revealed three distinct factors, enabling a nuanced examination of family routines before and during COVID-19, highlighted by significant changes in routine frequency and importance.

### Principal component analysis

Following the PCA of the pre-COVID data, we omitted four (of 28) items from the FRI during statistical processing because their factor loadings were lower than 0.30: Item 22, *At least one parent talks to their parents regularly*; Item 24, *Family checks in/out with each other when someone leaves or comes home*; Item 27, *The family almost always does certain things each time the children get out of line*; and Item 28, *Children do regular household chores*. The remaining 24 items were extracted into four distinct factors. Although Items 11, 17, and 20 showed some degree of cross-loading, we decided not to remove them. However, in our case, the decision not to remove Items 11, 17, and 20 was made based on statistical and theoretical grounds. Although these items showed some degree of cross-loading, the differences between their primary and secondary loadings exceeded the commonly accepted threshold of 0.10, and their primary loadings remained substantial and conceptually aligned with the intended factors. Additionally, the content of these items was theoretically meaningful and contributed to the internal consistency of their respective factors. Removing these items would have weakened the theoretical coherence and interpretability of the factors.

The revised PCA (without failed items) revealed a similar extraction into three distinct factors. Table 1 presents each factor’s final item loading, percentage of variance, and internal consistency for routine frequency. Factor 1 included 10 items related to family-member interactions inside the house environment; Factor 2 included nine items on interactions with the outside environment; and Factor 3 included five items about morning and evening general routines. Similar factors were calculated for the routine importance, and internal consistency was calculated for each (Factor 1 = 0.70; Factor 2 = 0.81; Factor 3 = 0.70). In this study, the KMO index was 0.87, and the Bartlett test of sphericity yielded *p* < 0.001, indicating suitability for factor analysis.

**Table 1:** Family routines inventory (frequency and importance) scores pre- and during-COVID. **Table 1** presents each factor’s final item loading, percentage of variance, and internal consistency for routine frequency. Factor 1 included ten items related to family-member interactions inside the house environment; Factor 2 included nine items on interactions with the outside environment; and Factor 3 included five items about morning and evening general routines.

Item		Factor 1	Factor 2	Factor 3
		Inside-family interactions	Outside-family interactions	Morning/evening routines
3	Working parent has a regular play time with the children after coming home from work.	**0.71**		
6	Parent(s) and children play together some time each day.	**0.66**		
10	Family has a certain "family time" each week when they do things together at home.	**0.66**		
1	Parent(s) have some time each day to talk with their children.	**0.62**		
8	The family has a “quiet time” each evening when everyone talks or plays quietly.	**0.61**	0.42	
4	Working parent takes care of the children some time almost every day.	**0.59**		0.46
21	The whole family eats dinner together almost every night.	**0.55**		
11	Parent(s) read or tell stories to the children almost every day.	**0.45**		0.40
20	At least some of the family eats breakfast together almost every morning.	**0.41**		0.41
12	Each child has some time each day to play alone.	**0.36**		
14	Young children go to play or school the same days each week.		**0.74**	
13	Children take part in regular activities after school.		**0.73**	
9	The family goes someplace special together each week.		**0.63**	
7	Nonworking parent and children do something together outside the home almost every day (*e.g*., shopping, walking)		**0.50**	
16	Parents have a certain hobby or sport they do together regularly.		**0.50**	
26	The family has certain things they almost always do to greet the working parent(s) at the end of the day.		**0.44**	
15	Children do their homework at the same time each day or night during the week.		**0.43**	
25	Working parent(s) come home from work at the same time each day.		**0.39**	
18	Children go to bed at the same time almost every night.			**0.72**
5	Children do the same things each morning as soon as they wake up.			**0.68**
2	Parents(s) have certain things they do every morning while getting ready to start the day.			**0.66**
19	The family eats at the same time each night.	0.40		**0.65**
17	Children have special things they do or ask for each night at bedtime (*e.g*., story, goodnight kiss, drink of water).	0.41		**0.49**

Factor				
Number of items		10	9	5
% of variance		18.00%	12.00%	6.42%
Internal consistency reliability (α)		0.78	0.75	0.70
Descriptive statistic	Range	1.20–4.00	1.00–4.00	1.20–4.00
	*M (SD)*	2.94 (0.56)	1.92 (0.06)	2.99 (0.66)

**Notes:**

Bolded values reflect items selected for each factor.

### FRI scale (Frequency and Importance) differences before and during COVID-19

Table 2 shows the differences in routine frequency and importance before and during COVID-19 (first lockdown), organized by the three new factors. The results indicated significant increases in the frequency and importance of items in Factor 1, inside family interaction. For example, parent(s) frequently had time each day to talk with their children (Item 1) before COVID-19 (*M* = 3.10, *SD* = 0.95) but, significantly, more time during COVID-19 (*M* = 3.29, *SD* = 0.89), *t*(252) = −3.80, *p* < 0.0, with no change in importance. The table also indicates significant decreases in the frequency and importance of items in Factor 2, outside family interactions, and Factor 3, morning and evening routines. For example, young children going to play or school on the same days each week (Item 14) decreased in frequency (pre-COVID: *M* = 2.70, *SD* = 1.07; during-COVID: *M* = 1.40, *SD* = 0.86), *t*(252) = 16.95, *p* < 0.01, and importance (pre-COVID: *M* = 2.40, *SD* = 0.71; during-COVID: *M* = 2.03, *SD* = 0.81), *t*(252) = 8.07, *p* < 0.01.

**Table 2:** Family routines inventory (frequency and importance) scores pre- and during-COVID. The table shows the differences in routine frequency and importance pre- and during-COVID (first lockdown), organized by the three new factors.

	Frequency							Importance						
	Pre		During		*t* (*df* = 252)	*p*	*d*	Pre		During		*t* (*df* = 252)	*p*	*d*
Item	*M*	*SD*	*M*	*SD*				*M*	*SD*	*M*	*SD*			
**Factor 1: Inside-family interactions**														
1	3.10	0.95	3.29	0.89	−3.80	<0.01	0.21	2.79	0.45	2.81	0.48	−0.63	0.53	0.04
3	2.71	0.93	3.20	0.84	−9.12	<0.01	0.55	2.75	0.48	2.81	0.45	−2.08	0.04	0.13
4	3.36	0.89	3.51	0.81	−3.09	0.02	0.18	2.74	0.53	2.79	0.46	−2.29	0.02	0.10
6	2.28	0.91	2.73	0.95	−8.66	<0.01	0.48	2.59	0.57	2.63	0.56	−1.31	0.19	0.07
8	2.10	1.0	2.43	1.08	−6.32	<0.01	0.32	2.41	0.68	2.44	0.67	−1.44	0.15	0.04
10	2.30	0.92	2.71	0.98	−7.79	<0.01	0.43	2.56	0.59	2.62	0.58	−2.25	0.02	0.10
11	2.66	1.09	2.83	1.08	−3.42	<0.01	0.16	2.55	0.63	2.55	0.64	0.00	0.99	0.01
12	2.99	1.0	3.14	1.02	−3.32	<0.01	0.15	2.54	0.58	2.56	0.59	−0.68	0.49	0.03
20	2.06	1.10	2.72	1.06	−9.10	<0.01	0.61	2.13	0.74	2.28	0.71	−3.78	<0.01	0.20
21	2.53	1.01	2.89	0.97	−7.17	<0.01	0.36	2.52	0.60	2.54	0.59	−0.66	0.51	0.03
**Factor 2: Outside-family interactions**														
7	2.20	0.81	2.11	0.99	1.76	0.08	0.10	2.20	0.69	2.14	0.75	1.74	0.08	0.08
9	2.04	0.71	1.77	1.01	4.42	<0.01	0.31	2.57	0.60	2.42	0.72	3.59	<0.01	0.23
13	2.36	0.88	1.61	0.92	12.64	<0.01	0.83	2.43	0.67	2.27	0.74	3.92	<0.01	0.23
14	2.70	1.07	1.40	0.86	16.95	<0.01	1.34	2.40	0.71	2.03	0.81	8.07	<0.01	0.48
15	2.42	1.16	2.25	1.16	3.02	<0.01	0.15	2.33	0.82	2.22	0.84	2.64	<0.01	0.13
16	2.13	0.88	2.00	1.06	2.16	0.03	0.13	2.62	0.59	2.48	0.71	3.90	<0.01	0.21
23	1.99	0.77	1.49	0.85	9.09	<0.01	0.62	2.42	0.63	2.39	0.74	0.98	0.33	0.04
25	2.94	1.00	2.43	1.20	6.66	<0.01	0.46	2.42	0.63	2.26	0.75	3.79	<0.01	0.23
26	2.48	1.18	2.27	1.19	3.99	<0.01	0.18	2.38	0.67	2.25	0.77	3.57	<0.01	0.18
**Factor 3: Morning/evening routines**														
2	3.57	0.78	2.93	1.04	9.15	<0.01	0.70	2.72	0.54	2.47	0.64	6.44	<0.01	0.42
5	3.48	0.68	2.97	0.92	8.46	<0.01	0.63	2.57	0.57	2.38	0.62	5.05	<0.01	0.33
17	3.49	0.87	3.49	0.90	−0.12	0.90	0.01	2.60	0.59	2.63	0.57	−1.15	0.25	0.05
18	3.35	0.71	2.74	1.06	9.73	<0.01	0.68	2.75	0.51	2.50	0.66	6.90	<0.01	0.42
19	2.96	0.91	2.82	0.97	3.07	<0.01	0.15	2.48	0.61	2.41	0.64	2.59	0.01	0.11
**Out of analysis:**														
22	3.16	0.89	3.35	0.82	−4.77	<0.01	0.22	2.71	0.56	2.76	0.51	−2.15	0.03	0.09
24	3.46	0.90	3.35	1.04	1.98	0.049	0.11	2.59	0.65	2.66	0.59	−2.54	0.01	0.11
27	2.81	1.01	2.85	1.05	−1.19	0.24	0.04	2.66	0.57	2.63	0.63	1.37	0.71	0.05
28	2.41	0.98	2.72	0.98	−6.40	<0.01	0.32	2.60	0.58	2.66	0.57	−1.79	0.07	0.10

### Routine factors and FQOL relationships during COVID-19

Table 3 depicts the significant positive moderate correlations between the three factors, frequency and importance, and FQOL during-COVID. However, parenting, family interactions, and material quality of life had no correlations with Factor 2 frequency.

**Table 3:** Means, standard deviations, and Pearson correlations for family routines inventory factors and family quality of life during-COVID. The table depicts the significant positive moderate correlations between the three factors, frequency and importance, and FQOL during-COVID.

	Factor	1	2	3	4	5	6	7	8	9	10
1	Factor 1 frequency										
2	Factor 2 frequency	0.25***									
3	Factor 3 frequency	0.48***	0.21**								
4	Factor 1 important	0.62***	0.10*	0.39***							
5	Factor 2 important	0.25***	0.38***	0.28***	0.54***						
6	Factor 3 important	0.31***	0.15*	0.60***	0.58***	0.57***					
7	Parenting	0.44***	0.07	0.33***	0.42***	0.23***	0.32***				
8	Family interaction	0.47***	0.01	0.32***	0.39***	0.17***	0.31***	0.79***			
9	Emotional	0.31***	0.17**	0.21**	0.28***	0.18**	0.25***	0.73***	0.72***		
10	Material	0.33***	0.05	0.33***	0.38***	0.15***	0.30***	0.69***	0.65***	0.65***	
11	Total well-being	0.45***	0.08	0.34***	0.42***	0.21***	0.34***	0.91***	0.91***	0.86***	0.84***

**Notes:**

* *p* < 0.05.

** *p* < 0.01.

*** *p* < 0.001.

Stepwise regression analyses conducted to predict total family well-being by the routines’ frequency and importance factors (Table 4) showed that Factor 1 frequency predicted 20% of the variance (R2 = 0.19, *p* < 0.001) and Factor 3 importance predicted 4% more (R2 = 0.23, *p* < 0.001).

**Table 4:** Standardized coefficients predicting total well-being during-COVID. The table shows that Factor 1 frequency predicted 20% of the variance and Factor 3 importance predicted 4% more.

Variable	B	*SE* B	ß	B	*SE* B	ß
Factor 1 routine	0.60	0.07	0.45***	0.51	0.08	0.38***
Factor 3 important				0.40	0.10	0.22***
*R*^2^ (adjusted)	0.20 (0.19)			0.24 (0.23)		
*F*	62.15***			39.77***		

**Notes:**

*SE*, standard error.

*** *p* < 0.001.

## Discussion

Our study reveals significant changes in family routines during the COVID-19 pandemic. The findings indicate that changes in the frequency and perceived importance of family routines during the lockdown were associated with reported levels of FQOL, particularly in areas related to family interaction and parenting. These findings resonate with existing research during the COVID-19 pandemic (*e.g*., Carroll et al., 2020). For instance, Prime, Wade & Browne (2020) highlighted a dramatic shift in family routines during the pandemic, changes not witnessed since World War II. Recognizing a new and unfamiliar reality prompted us to analyze family routine changes from a fresh perspective.

Globally, the COVID-19 pandemic thrust families into an unprecedented and unfamiliar situation, compelling them to confront a starkly different reality in their daily lives than any they had previously known. Numerous activities previously conducted outside the home were suddenly canceled or incorporated into the family’s daily life, requiring parents to adapt their family routines to reduce external interactions. This transition entailed shifting from traditional work, educational, and social settings to a predominantly home-based existence, significantly affecting family functioning. Our study represents one of the empirical investigations into family routines during the COVID-19 pandemic, encompassing an analysis of alterations in routine frequency and importance as parents self-reported. The study’s significant and unique contribution is treating the family as a unit (rather than as separate family members) in examining relationships between family daily routines and FQOL.

### FRI scale (Frequency and Importance) changes before and during COVID-19

The PCA provided insights into the underlying structure of family routines as assessed by the FRI. This analysis led to extracting three distinct factors, shedding light on different facets of family routines. Factor 1 highlights interactions within the family, mainly at home, focusing on communication, bonding, and activities such as talking, playing, and reading, along with established times for joint activities, thereby prioritizing quality time and shared experiences. Factor 2 involves external interactions, including family outings and scheduling children’s activities, linking to educational institutions and community centers. Factor 3 emphasizes structured routines, particularly morning and bedtime activities, promoting regular sleep patterns and shared mealtimes.

The COVID-19 pandemic fundamentally altered parental realities, presenting a scenario akin to an extended vacation but with unique challenges such as distance learning, limited outdoor activities, and suspended social interactions. This shift necessitates parental guidance to navigate an uncertain period without a defined end, diverging significantly from the predictable breaks of weekends or holidays (Brazendale et al., 2017). This ongoing uncertainty underscores the need for adaptable family routines in managing the new daily norms.

Our study identified a significant uptick in routines categorized under Factor 1, reflecting enhanced family connections during the home quarantine phase, in line with global observations (Günther-Bel et al., 2020; The Royal Children’s Hospital Melbourne, 2020; Tang et al., 2021). However, this increase also brought about heightened parental stress due to prolonged homestays and intensified family communication (Adams et al., 2021; Bates et al., 2021). Conversely, routines under Factors 2 and 3 declined, mirroring the reduced access to external resources and shifting the home into the core of family life (Evans et al., 2020; Sorbring et al., 2022), which underscores the evolving nature of family dynamics amid the pandemic.

### Routine factors and FQOL relationships

Earlier research mentioned the relationships between family routines and FQOL (*e.g*., Rodger & Umaibalan, 2011; Schaaf et al., 2011). However, our study found different relationships between family routines and FQOL by looking at associations for each factor separately. Our results shed light on family functioning during a global pandemic and important routines associated with better FQOL in COVID-19—a time of acute health, economic, and social stress. Specifically, the literature showed that confinement, lack of freedom, and loss of usual routines are consequences perceived as highly distressing in similar pandemic situations (*e.g*., Braunack-Mayer et al., 2013; Hawryluck et al., 2004).

This finding makes sense, given the high demands on parents during crises like the pandemic. These include balancing work and childcare responsibilities and providing children with a stable and secure environment (Fong & Iarocci, 2020). Thus, concerns over potential job loss and arranging childcare might contribute to increased stress and decreased well-being. Previous studies highlighted the importance of routines and rituals as valuable resources for FQOL and overall health and as protective factors against significant adversity (Crespo et al., 2013). Specific types of routines, like bedtime routines, have been closely studied for their impact on family dynamics and child development. Consistent bedtime routines have been associated with better sleep outcomes, improved parent–child relationships, and enhanced emotional well-being (Mindell et al., 2009). During the COVID-19 pandemic, the blurring boundaries between work, school, and home life challenged maintaining such routines. However, families that successfully preserved or adapted their bedtime routines reported higher family functioning and well-being (Bates et al., 2021).

Those relationships between family routines and FQOL have been explored through resilience theory, which posits that routines can help families bounce back from adversity (Walsh, 2016). Brown et al.’s (2020) study found that during the early months of the COVID-19 pandemic, families who maintained structured daily routines experienced higher FQOL and lower stress levels. These findings suggest that routines can buffer the negative impacts of crises on family well-being and may even be beneficial during disasters. However, little evidence has documented whether family routines exert similar protective influences on children’s and families’ mental health in such crises (Glynn et al., 2021).

Family routines may represent a promotive factor because of their associations with resilience in low- and high-risk conditions (Masten & Narayan, 2012). Glynn et al. (2021) hypothesized that families would find positive external influences even during the COVID-19 pandemic’s distinct circumstances. Practicing family routines predicted better child mental health, and this protective effect persisted even amid covarying income, dual-parent status, and food insecurity with maternal depression and stress. They highlighted that adhering to family routines helps maintain a predictable, structured home environment and may mitigate these adverse effects. This study adds to existing literature by treating the family as a dynamic unit and situating routines within their broader cultural and ecological context.

### Predicting FQOL

Our research demonstrates that daily routine factors predict 24% of FQOL variance during the COVID-19 pandemic, highlighting the critical role of maintaining routines for family well-being (Algahtani et al., 2021; Engel-Yeger & Engel, 2023). Prior studies, including that of Ali & Malik (2015), identified family functioning and health behaviors as significant FQOL predictors, reinforcing the importance of routines in adaptive coping and effective parenting (Dunn et al., 2000; McCubbin & McCubbin, 1988). Loss of routine significantly affects FQOL, as shown in health care workers during the pandemic (Woon et al., 2021). Despite our hypothesis, maintaining routines correlated with—rather than predicted—positive parenting, suggesting its role as an outcome of engaged parenting practices (Koblinsky, Kuvalanka & Randolph, 2006). The pandemic underscored the need for adaptable routines to support family resilience and FQOL (Brown et al., 2020; Carrion, Woods & Massey, 2020; Lawson, Piel & Simon, 2020), with structured routines vital for emotional and psychological development, especially in children.

## Conclusions

This study explored and discussed changing family routines and their relationship to FQOL. It offers two major contributions. The first is classifying routines into three factors essential to the daily changes the COVID-19 crisis brought about. The second is the ability to predict factors that family intervention processes may address in the short term during the crisis and in the long term as pre-crisis prevention to strengthen families’ resilience and preserve their FQOL in times of crisis.

This study also provides insights into COVID-19’s impact on family well-being, dynamics, and parenting in families with underage children and factors that explain differences across families in adapting to changing circumstances. By examining routine changes and their associations with family well-being, this research aids in understanding how families adapt and cope during challenging times. Such insights are essential for policymakers and parents seeking to navigate their families through crises effectively. Specifically, families can leverage these insights to prioritize routines that strengthen their well-being during crises. Furthermore, policymakers can use this evidence to develop targeted recommendations and guidelines supporting beneficial family routines during future disruptions. Finally, practitioners could use these results to design focused interventions that enhance family resilience and adaptive capacities under stressful circumstances.

This study has limitations, including potential bias from using a convenience sample with possibly higher socioeconomic and education levels and reliance on self-reported pre-COVID-19 data, which might influence responses. The questionnaires were designed pre-crisis and were lengthy, along with the rapid research design post-outbreak, which may affect accuracy. Future studies should aim for diverse, longitudinal samples and consider these design challenges. Additionally, the current study relies solely on quantitative self-report surveys, limiting our ability to deeply capture nuanced family experiences and coping strategies. Future research incorporating qualitative methods, such as interviews or focus groups, could provide richer context and a deeper understanding of how families experienced routine changes and adapted their coping mechanisms during the COVID-19 lockdown. Furthermore, future studies should examine family routines and FQOL across different cultural and socioeconomic groups to strengthen the generalizability of these findings and should examine these associations longitudinally and across diverse populations to explore the long-term impact of routine disruptions. Practitioners should consider integrating family routine assessment into support programs for families during crises.

## Supplemental Information

10.7717/peerj.19587/supp-1 Supplemental Information 1 COVID-19 Raw Data.

10.7717/peerj.19587/supp-2 Supplemental Information 2 COVID-19 Syntax.

10.7717/peerj.19587/supp-3 Supplemental Information 3 Codebook for raw data.

10.7717/peerj.19587/supp-4 Supplemental Information 4 STROBE checklist.

## Additional Information and Declarations

### Data Availability

The following information was supplied regarding data availability:

The raw data and the syntax are available in the Supplemental Files.
