# Supplementary material for: Exploring changes in family routines and family quality of life among Israeli families during the COVID-19 pandemic
Source: PeerJ. 2025 Jul 3;13:e19587. doi: 10.7717/peerj.19587 (PMC12229148; doi:10.7717/peerj.19587)
Supplement: Supplemental Information 3 [file peerj-13-19587-s003.pdf]

Categorical Data

Line	Variable name	Values
Line 1	No	
Line 2	Parent 1 age	
Line 3	Parent 2 age	
Line 4	Role (Who fulfills the questionnaire)	1. Mother
		2. Father
Line 5	Education (for the parent who fulfills the questionnaire)	1. High school
		2. Vocational
		3. Academic
Line 6	Income	1. Above the average monthly salary
		2. Below the average monthly salary
Line 7	Place of Residence	1. City
Line 8	No. of children	2. Other
Line 9-36	FRI_routineB_Q1-Q28	FRI Routine items before the COVID-19
Line 37-64	FRI_importentB_Q1-Q28	FRI Important items before the COVID-19
Line 65-92	FRI_routineK_Q1-Q28	FRI Routine items during the COVID-19
Line 93-120	FRI_importentK_Q1-Q28	FRI Important items during the COVID-19
Line 121	FRI_routine_B	FRI Routine sum of the items before the COVID-19
Line 122	FRI_routine_K	FRI Routine sum of the items during the COVID-19
Line 123	FRI_importent_B	FRI important sum of the items before the COVID-19
Lune 124	FRI_importent_K	FRI important sum of the items during the COVID-19
Line 125	FRI_R_1_K	Factor 1 routine: Inside-family interactions
Line 126	FRI_R_2_K	Factor 2 routine: Outside- family interactions
Line 127	FRI_R_3_K	Factor 3 routine: Morning/ evening routines
Line 128	FRI_I_1_K	Factor 1 important: Inside-family interactions
Line 129	FRI_I_2_K	Factor 2 important: Outside- family interactions
Line 130	FRI_I_3_K	Factor 3 important: Morning/ evening routines
Line 131-151	FQL_B_Q1-Q21	FQL – 21 items before the COVID-19
Line 152-172	FQL_K_Q1-Q21	FQL – 21 items during the COVID-19
Line 173	FQL_B_familiy_interaction_M	Mean of items: 1, 7, 10–12, and 18 before the COVID-19
Line 174	FQL_B_Parenting_M	Mean of items: 2, 5, 8, 14, 17, and 19 before the COVID-19
Line 175	FQL_B_emotional_wellbeing_M	Mean of items 3, 4, 9, and 13 before the COVID-19
Line 176	FQL_B_physical_material_wellbeing_M	Mean of items 6, 15, 16, 20, and 21 before the COVID-19
Line 177	FQL_B_total_wellbeing_M	The mean of 21 items before the COVID-19
Line 178	FQL_K_familiy_interaction_M	Mean of items: 1, 7, 10–12, and 18 during the COVID-19
Line 179	FQL_K_Parenting_M	Mean of items: 2, 5, 8, 14, 17, and 19 during the COVID-19
Line 180	FQL_K_emotional_wellbeing_M	Mean of items 3, 4, 9, and 13 during the COVID-19
Line 181	FQL_K_physical_material_wellbeing_M	Mean of items 6, 15, 16, 20, and 21 during the COVID-19
Line 182	FQL_K_total_wellbeing_M	The mean of 21 items during the COVID-19
